# Effect of Endolymphatic Hydrops on Sound Transmission in Live Guinea Pigs Measured with a Laser Doppler Vibrometer

**DOI:** 10.1155/2016/8648297

**Published:** 2016-12-14

**Authors:** Chen-Ru Ding, Xin-Da Xu, Xin-Wei Wang, Xian-Hao Jia, Xiang Cheng, Xiang Liu, Lin Yang, Bu-Sheng Tong, Fang-Lu Chi, Dong-Dong Ren

**Affiliations:** ^1^Department of Otology and Skull Base Surgery, Eye Ear Nose & Throat Hospital, Fudan University, Shanghai 200031, China; ^2^Shanghai Auditory Medical Center, Shanghai, China; ^3^Key Laboratory of Hearing Science, Ministry of Health, Shanghai, China; ^4^Research Center, Eye Ear Nose & Throat Hospital, Fudan University, Shanghai 200031, China; ^5^The First Affiliated Hospital of AnHui Medical University, Hefei, China

## Abstract

*Objective*. This study aimed at describing the mechanism of hearing loss in low frequency and the different dynamic behavior of the umbo, the stapes head, and the round window membrane (RWM) between normal guinea pigs and those with endolymphatic hydrops (EH), using a laser Doppler vibrometer (LDV).* Methods*. Cochlear sections were stained with hematoxylin and eosin (HE) to evaluate the hydropic ratio (HR). Auditory brainstem responses (ABR) and whole-mount immunostaining were measured. Displacement of the umbo, stapes head, and RWM in response to ear-canal sound was evaluated using a LDV.* Results*. Mean HR values in EH model of all the turns are larger than the control group. The ABR threshold of the EH group was significantly higher than that of the control. Strong positive correlation was found between HR at apical turn and ABR threshold elevation at 1000 Hz and at subapical turn and ABR threshold elevation at 2000 Hz. FITC-phalloidin immunostaining of the cochlear basilar membrane in the apical, subapical, and suprabasal turns showed missing and derangement stereocilia of third-row outer hair cells. The umbo, stapes head, and RWM displacement in ears with EH was generally lower than that of normal ears. The EH-induced differences in stapes head and RWM motion were significant at 0.5 kHz.* Conclusion*. The LDV results suggested that the higher inner ear impedance in EH affected the dynamic behavior of the two opening windows of the cochlea and then reduced the vibration of the ossicular chain by increasing the afterload, resulting in acoustic dysfunction. The vibration reduction mainly occurred at low frequencies, which has related with the morphology changes of the apical and subapical turns in EH model.

## 1. Introduction

In mammals, sound waves stimulate the cochlea via the vibration of the ossicular chain. The opposite vibrating phase between the round window and the oval window causes relative motion between the endolymph and perilymph and thus produces displacement waves travelling on the spirally basilar membrane. The motion of hair cell stereocilia created by basilar membrane (BM) vibration gates stereocilia transduction channels, leading to the generation of hair cell receptor potential and the excitation of afferent auditory nerve fibers [1]. Changes in cochlear lymphatic fluid homeostasis may result in cochlear acoustic dysfunctions, like endolymphatic hydrops (EH), semicircular canal dehiscence, labyrinthine fistulas, and so forth [2, 3].

Since Hallpike and Carins [4] described the presence of EH in the temporal bones of patients with Ménière's Disease (MD) in 1938, EH has generally been accepted as the basic histopathologic sign of this disease, which is an intractable disease that results in hearing loss that is often fluctuating and initially involves the low frequencies [5]. Wu et al. [6] reported that low-tone and middle-tone hearing thresholds were related to the severity of EH in the cochlea. In the study by Lee et al. [3], spontaneous low frequency air-bone gaps in evaluating hearing sensitivity were found in approximately 13.9% of patients with Ménière's Disease and may indirectly reflect aggravation of the EH in the cochlear and the vestibular compartments. Yoshida et al. [7] suggested an association between endolymphatic hydrops and low frequency hearing loss in a 13-year-old girl with mutation of the SLC26A4 gene. Endolymphatic hydropic condition was described in certain cases of sudden deafness [8, 9]. Noguchi et al. [10] assumed that EH give rise to the pathogenesis of acute low-tone sensorineural hearing loss (ALHL) with little or no impairment of hair cells that resembles early-stage MD. It is widely hypothesized that the hydrops generates the clinical symptoms of this illness. Yet, there are few studies that have investigated how the dilated endolymph affects the middle ear acoustic transmission.

A laser Doppler vibrometer (LDV) is a noncontact, established optical technique that can be used to measure the displacement of middle ear components in response to sound stimulation [11–16]. It uses the Doppler-shift principle to determine the instantaneous velocity of a moving object by comparing the frequency of the laser's emitted light with the frequency of the light reflected from the moving object. This technique has been used to test the vibration of the round window membrane (RWM), the tympanic membrane (TM), and the stapes footplate in fresh and embalmed cadaveric human temporal bone and animal specimens. Moreover, animal models of different diseases have been created to investigate vibration changes in the ossicular chain and RWM, to explore the potential mechanism underlying the clinical symptoms of diseases. For instance, vibration of the RWM that is associated with acute otitis media is significantly decreased compared with those of the RWM from the ears of normal guinea pigs [15]. Moreover, middle ear effusion reduces the mobility of the TM, the incus tip, and the RWM at frequencies above 1 kHz in guinea pigs [17]. The mechanical properties of the incudostapedial joint directly affect the stapes movement or the middle ear transfer function for sound transmission [18]. However, no prior study has reported changes in the dynamic behavior of the umbo, the stapes head, and the RWM in association with EH.

To better understand how EH affects the sound transmission process and hearing loss in low frequency, we used guinea pigs to create EH models and measured the hydropic ratio (HR) and morphology in each turn, the vibration of the umbo, stapes head, and RWM, as well as the auditory-evoked brainstem response (ABR). The primary objective of this study was to compare the different dynamic properties of the ossicular chain and the RWM between normal and EH guinea pigs and explore the potential pathogenic mechanism underlying the associated hearing loss at low frequencies.

## 2. Material and Methods

### 2.1. Animals

All animal work conducted during the course of this study was approved by the institutional animal care and use committee at Eye Ear Nose & Throat Hospital, Fudan University, and conformed to the National Institutes of Health guide for care and use of laboratory animals.

Eighteen healthy albino male guinea pigs with an initial weight of 250 g–300 g and a positive Preyer reflex were used in this study. All animals were free of middle ear diseases, such as tympanic membrane perforation or otitis media, as evaluated by otoscopic examination. Identification of preexisting abnormalities of auditory function was made by a prerecruiting ABR measurement in each animal. If an abnormal response was found, the animal was excluded from the study. Guinea pigs were randomly allocated into two groups, the control group (9 guinea pigs with sham surgical procedure in the right ear) and the EH group (9 guinea pigs with obliteration of the endolymphatic sac in the right ear). Animals in the two groups were raised for 8 weeks postoperatively. Specimens from each group (control and EH) were used for histologic observations of paraffin sections (six ears) and whole-mount immunostaining (three ears).

### 2.2. Surgical Procedure

#### 2.2.1. Creation of EH Model

The right ears of the nine animals in the EH group were prepared. The obliteration of the endolymphatic sac was performed surgically using an extradural posterior cranial fossa approach, under sterile conditions and under a surgical microscope (6030116204, Carl Zeiss, Jena, Germany). The right ears of the nine animals in the control group were subjected to sham operation. Exposure of the sigmoid sinus through the occipital bone was accomplished without obliteration of the endolymphatic sac. The temperature was maintained stably to keep animals warm during surgery, by using an electric heating pad. Anesthesia was induced with a combination of ketamine hydrochloride (35 mg/kg, intramuscular injection) and 1% xylazine hydrochloride (10 mg/kg, subcutaneous injection). Additional anesthesia was administered as required to maintain areflexia.

#### 2.2.2. Preparation of the Umbo, Stapes Head, and RWM for LDV Measurement

**Figure 1:**
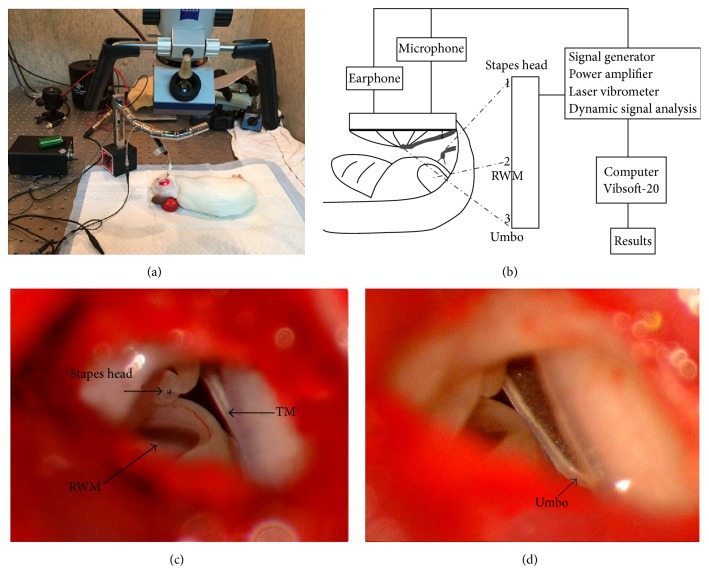
(a) the measurement system included a Compact Laser Vibrometer coupled with a microscope with a micromanipulator, signal generator, and power amplifier. (b) The schematic diagram of the laser Doppler vibrometer (LDV) detecting system. (c and d) The incus-stapes complex, round window membrane, and the medial side of the umbo, viewed from the opened middle ear cavity. The laser reflective tapes were placed at the location marked with an *∗* ((c) stapes head and middle of the RWM; (d) umbo).

The right ears from six animals in the control group as well as those from the EH group were prepared. After anesthesia (as also described above), the right auricles were cut off and the dorsal auditory bulla was opened into a square block under a surgical microscope (6030116204, Carl Zeiss, Jena, Germany), from which the incus-stapes complex, RWM, and the medial side of the umbo could be viewed clearly (Figure 1). A 0.25–0.5-mm^2^ square (approximately 2–4 pieces, each piece with a mass of 40 *μ*g) of laser reflective tape (3 M, Maplewood, MS, USA) was placed on the medial side of the umbo, the stapes head, and the middle of the RWM, as the laser-reflecting target (Figures 1(a)–1(d)). The mass of these pieces was deemed small enough not to affect the measurement. Membrane structures were not damaged during the preparation. Hemorrhagic spots were remedied through unipolar electrocoagulation.

### 2.3. ABR Measurement

ABR measurements in the control group and in the 8-week EH group were performed before the surgery and prior to the LDV measurement. After anesthesia, the ABR (Bio-Logic NAVPRO, 580-NAVPR2, Natus Medical Incorporated, Pleasanton, CA, USA) were tested in a sound-proofed booth to assess the auditory threshold. Needle electrodes were placed subcutaneously at the vertex for recording and behind the bilateral ears as reference and ground electrodes. Stimulation was presented as tone bursts (5 ms duration, 0.5 ms rise-fall time, Blackman envelope) at a frequency of 0.5, 1, 2, 4, 6, and 8 kHz; the sound-intensity level was decreased in 10 dB steps from 80 to 20 dB SPL; 500 responses at each sound level were recorded and averaged. If I, III, and V waves disappeared, we increased the tone bursts by 5 dB repeatedly to judge the threshold by the waveform.

### 2.4. LDV Measurement

The measurement system included a Compact Laser Vibrometer (CLV-2534-4, Polytec, Wurzburg, Germany) coupled with a microscope (OPMI 1-FC, Carl Zeiss, Jena, Germany) with a micromanipulator (A-HLV-MM30, Polytec, Wurzburg, Germany), signal generator (33210A, Agilent, Santa Clara, CA, USA), and power amplifier (RMX 850, QSC, Costa Mesa, CA, USA) (Figure 1(a)). The intensity of each excitation frequency was calibrated to 85 dB SPL using a sound-level meter (AWA-5661-1B, AiHua, Yiyang City, China). An earphone associated with the microphone (ER-4PT, ER-7C, HLV-SPEC Adapter, Etymotic, Elk Grove Village, IL, USA) was inserted into the osseous external auditory canal to give signal stimuli and monitor sound pressure. The distance between the tympanic membrane and the earphone and microphone was maintained at 1 mm. The vibration of the moving surface was acquired through the reflective bead by the system and recorded on computer software (Vibsoft-20, Polytec, Wurzburg, Germany) for further analysis. The vibration amplitude of the moving surface was calculated from the voltage output of the laser vibrometer's velocity decoder. Appropriate anesthesia (subcutaneous injection of 10 mg/kg xylazine hydrochloride 1 h after normal anesthesia described above) was maintained to retain respiratory amplitude so as not to affect the test during the process. Testing was conducted in a sound-proofed booth to maintain a high signal-to-noise ratio. For each stimulus frequency, sound stimuli were repeated three times with a good signal-to-noise ratio, after which all data were averaged.

### 2.5. Section Processing

Six animals of each group were sacrificed by an overdose of anesthetic; intracardiac perfusion was performed with 150 mL of 0.2 M PBS, followed by 4% polymerized formaldehyde, and the temporal bones were then removed and fixed in 4% polymerized formaldehyde (pH = 7.4) for more than 24 h at 4°C. The temporal bones were decalcified in ethylenediaminetetraacetic acid, dehydrated in increasingly higher concentrations of alcohol, embedded in paraffin, and sectioned serially at 10 *μ*m in the plane parallel to the modiolus. Cochlear sections were stained with hematoxylin and eosin (HE) and then observed under a light microscope (6030116204, Carl Zeiss, Jena, Germany).

### 2.6. Whole-Mount Immunostaining

Three guinea pigs of each group were killed by means of decapitation by overdose anesthesia. Under magnification, the tympanic bullae were dissected from the surrounding tissues; the bony wall of the cochlea was removed with a pick and forceps to expose the upper aspect of the organ of Corti. Cochleae were fixed in a 4% paraformaldehyde (PFA) in phosphate-buffered saline (PBS) for 24 hours and then we dissected the cochlea and get the basilar membrane of the cochlea. The whole-mount tissues were incubated with Alexa Fluor 488-conjugated phalloidin (Invitrogen, USA, 1 : 1000) for 30 minutes to stain for F-actin prior to mounting. Confocal fluorescent microscope (Zeiss, LSM800, Germany) was used in scanning the surface image of Corti stained for stereocilia with green phalloidin.

### 2.7. Quantification of the Hydropic Ratio (HR) of the EH Models

In order to quantify the hydropic ratio (HR) of the EH models, a proportional measurement was conducted. Areas of scala media (SM) and scala vestibule (SV) (Figure 3(a)) were first obtained by using Photoshop CS6 and scala media area ratio (SMR) was calculated in formula

(1) $$\begin{array}{c} SMR=\frac{{SM}_{area}}{{SM}_{area}+{SV}_{area}}. \end{array}$$

As deviations in the plane of section and interanimal variability in anatomy cannot be avoided exactly, SMR is imprecise for representing hydropic degree of EH animals. Then we use the ratio of SMR in the EH ear divided by SMR in the contralateral ear to quantify the hydropic degree in the EH group, whose right ears were subject to obliteration of the endolymphatic sac. And this ratio was named as hydropic ratio (HR) here. In formula

(2) $$\begin{array}{c} HR=\frac{\text{SMR in the EH ear}}{\text{SMR in the contralateral ear}}\\ \text{a HR value of one suggests no hydrops in the given turn}. \end{array}$$

This is when hydropic ratio is close to 1; that to say, there is no hydrops in the given turn.

### 2.8. Statistical Analysis

Data are presented as mean ± standard error. All analysis was performed using the SPSS 19.0 statistical package. Two-tailed Students' *t*-tests were used to determine the confidence interval for comparison between two groups and *p* values ≤ 0.05 were considered significant. Spearman bivariate correlation analysis was taken to explore the relationship between ABR threshold elevation and endolymphatic hydropic ratio.

## 3. Results

### 3.1. Observation of Tissue Sections of the Cochlea in Ears with EH

**Figure 2:**
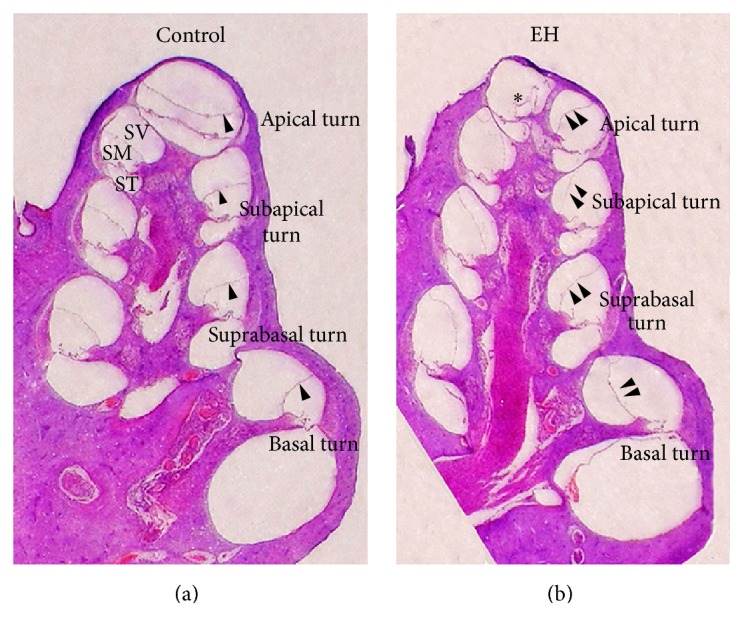
Mid-modiolar hematoxylin and eosin-stained section of the cochlea in the right ears of the control group (a) and the 8-week endolymphatic hydrops (EH) group (b). SV: scala vestibuli; SM: scala media; ST: scala tympani; arrowhead: Reissner's membrane in the control group; double arrowheads: distention of Reissner's membrane in the 8-week EH group; *∗*: rupture of Reissner's membrane due to artifact in the apical turn.

The mid-modiolar HE-stained section of the cochlea showed that there was no displacement of Reissner's membrane in the control ear and the angle between Reissner's membrane and the osseous spiral lamina was almost 45°, suggesting that there was no EH in the control group (Figure 2(a)).  Figure 2(b) illustrates that Reissner's membrane of each turn bulged significantly towards the scala vestibule in the 8-week EH group. Reissner's membrane was attached to the bony wall of the scala vestibule (SV) in the subapical turn of the cochlea. A high level EH was observed in the subapical turn, while the EH in the basal turn was markedly more moderate. These results agreed with those from a previous study by Chi and Liang [19] and indicated that the chronic EH model was successfully created.

### 3.2. Quantification of the Hydropic Ratio (HR) of the Four Turns in EH Models

**Figure 3:**
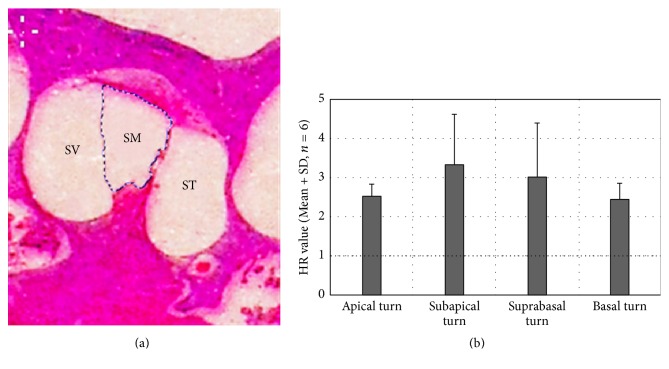
Mean HR value of each turn in the EH models. (a) HR was measured by the area of SM and SV. SM means the scala media, SV means scala vestibule, and ST means scala tympani. (b) Subapical turn had developed the most prominent hydrops with a mean HR value of 3.34, secondly followed by suprabasal turn. Mean HR values of all the turn are larger than 1 (dotted line: an indicator of no EH existence).

In order to observe the extent of EH, we had quantified the HR in each turn of the cochlea. When hydropic ratio is close to 1, there is no labyrinthine hydrops in the given turn. The HR is much larger according to the most serious hydrops in the scala media. The results of the individual and average HR for each turn of all animals in the EH group are shown at Table 1 and Figure 3(b). HR values for all turns of the six EH models were obviously larger than 1 (*p* < 0.05), demonstrating that conspicuous hydrops was induced. The most serious labyrinthine hydrops is in the subapical turn, and the second is in the suprabasal turn.

### 3.3. Effect of EH on ABR Measurement

**Figure 4:**
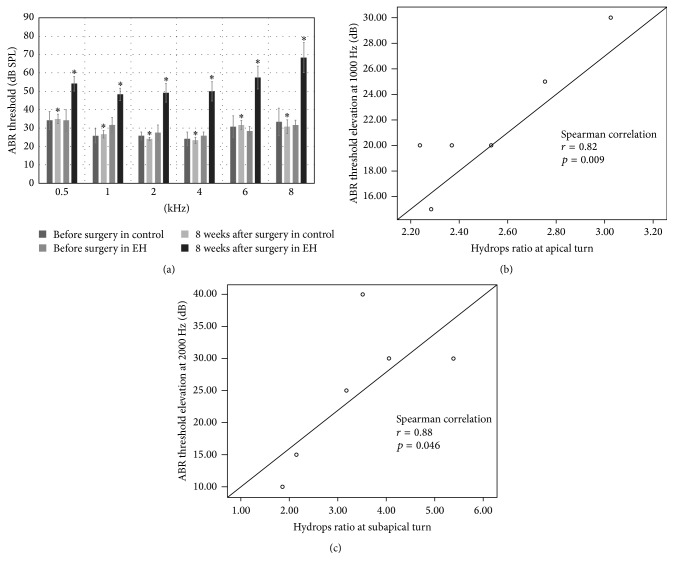
(a) The mean thresholds of ABR were recorded before surgery and 8 weeks after surgery in control and EH group. The mean ABR threshold of the EH cases was significantly higher than that of the control group at 0.5, 1, 2, 4, 6, and 8 kHz (^*∗*^ *p* < 0.05) 8 weeks after surgery. (b) Correlation between HR at apical turn and ABR threshold elevation at 1 kHz. (c) Correlation between HR at subapical turn and ABR threshold elevation at 2 kHz.

The auditory threshold of guinea pigs was assessed from the ABR threshold. The results of the ABR threshold in the control group and EH group are listed in Table 2 and Figure 4(a). Both the mean and standard deviation of each group were recorded. Elevation of the ABR threshold was observed in ears associated with EH. The ABR threshold was elevated in the EH group relative to the control group by more than 25 dB at 2, 4, 6, and 8 kHz and by less than 25 dB at 0.5 and 1 kHz. Student's *t*-tests revealed that the mean ABR threshold of the EH cases was significantly higher than that of the control group at 0.5, 1, 2, 4, 6, and 8 kHz (*p* < 0.05).

Spearman bivariate correlation analysis was taken to explore the relationship between ABR threshold elevation and extent of the endolymphatic hydrops. Strong positive correlation was found between HR at apical turn and ABR threshold elevation at 1000 Hz (*r* = 0.82, correlation is significant at the 0.01 level), as well as between HR at subapical turn and ABR threshold elevation at 2000 Hz (*r* = 0.88, correlation is significant at the 0.05 level). No significant correlations were found among the remainder. Results were presented in Figures 4(b) and 4(c).

### 3.4. Effect of EH on Movement of Umbo, Stapes Head, and RWM

**Figure 5:**
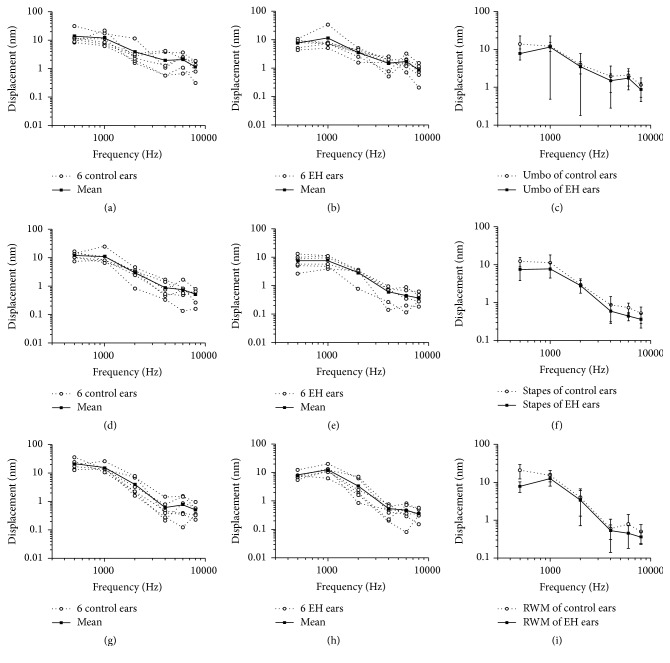
Peak-to-peak displacement-frequency curve of the umbo (a–c), stapes head (d–f), and round window membrane (RWM) (g–i) in response to 85 dB SPL sound stimuli at the ear canal. (a) Six individuals and their mean value in the control group (dotted line for the individuals and solid line for the mean value). (b) Six individuals and their mean value in the endolymphatic hydrops (EH) group (dotted line for the individuals and solid line for the mean value). (c) Mean ± SD (*n* = 6) of the control and EH groups, respectively: dotted line for the control group and solid line for the EH group. (d) Six individuals and their mean value in the control group (dotted line for the individuals and solid line for the mean value). (e) Six individuals and their mean value in the endolymphatic hydrops (EH) group (dotted line for the individuals and solid line for the mean value). (f) Mean ± SD (*n* = 6) of the control and EH groups (dotted line for the control group and solid line for the EH group). (g) Six individuals and their mean value in the control group (dotted line for the individuals and solid line for the mean value). (h) Six individuals and their mean value in the endolymphatic hydrops (EH) group (dotted line for the individuals and solid line for the mean value). (i) Mean ± SD (*n* = 6) of the control and EH groups (dotted line for the control group and solid line for the EH group).

The LDV measurements showed clear differences between control and EH ears. These differences are shown in Figure 5. Figures 5(a) and 5(b) illustrate the peak-to-peak displacement amplitude-frequency curves of the umbo from the six control ears and that of the six EH ears, respectively, over the 0.5–8-kHz range in response to 85 dB SPL stimuli in the ear canal. The two groups possessed similar displacement-frequency curves for the 0.5–8-kHz range and the results of the EH ears were lower than that for the control ears overall (Figure 5(c)). A maximum displacement amplitude, almost 14 nm, presented at 0.5 kHz in the control group. In the EH group, peak displacement was found at 1 kHz (11.48 nm) and there was a sudden decrease in displacement amplitude between 1 and 2 kHz frequencies in both groups. Mean ± SD (*n* = 6) of the control and EH groups was compared in Figure 5(c) showing that there are no significant differences in each frequency; particularly the biggest reduction of displacement amplitude among the two groups was present at 0.5 kHz (6.05 nm, *p* = 0.12) and this reduction remained below 1 nm at frequencies above or equal to 1 kHz.

Figures 5(d) and 5(e) display the peak-to-peak displacement curves of the stapes head in two groups. Displacements in the EH group were generally lower than those in the control group (Figure 5(f)). The displacement amplitude reached a maximum of 12.3 nm at 500 Hz and decreased gradually to 0.52 nm at 8 kHz in normal ears, while in the EH group the maximal displacement amplitude was 7.6 nm at 1 kHz. A statistically significant difference (*p* < 0.05) was found at 500 Hz and the reduction reached almost 4.82 nm.

Figures 5(g) and 5(h) show the peak-to-peak displacement curves of the RWM in both groups. Each curve shows a prominent displacement peak at 0.5, 1, 2, 4, 6, and 8 kHz. The displacement decreased along with the increase in frequency from 0.5 to 8 kHz in the two groups in response to 85 dB SPL input at the ear canal. The displacements of the EH group were generally lower than those in the control group (Figure 5(i)). There was a statistically significant difference (*p* < 0.05) at 0.5 and 6 kHz. For RMW, the best vibration response to displacement in the control ears was 20.79 nm at 500 Hz, while peak displacement in the EH group was 12.43 nm at 1 kHz.

**Figure 6:**
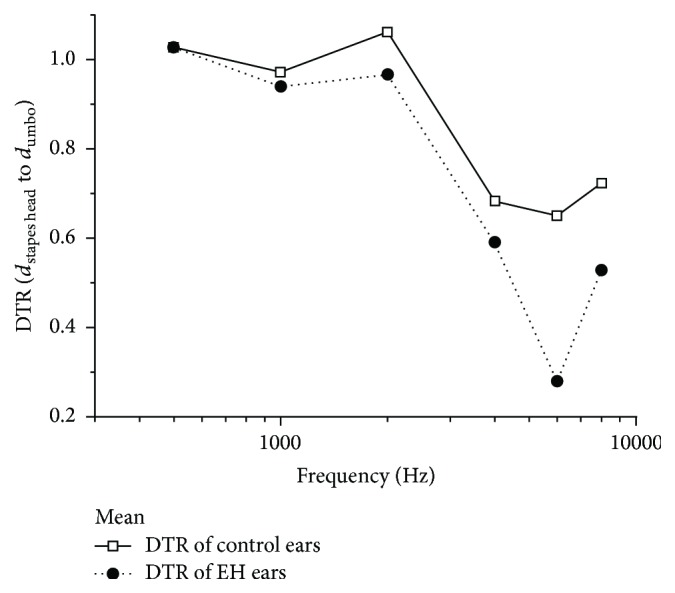
Displacement transmission ratio (DTR) of the stapes head to the umbo (mean value, *n* = 6) in control ears (solid line), and ears with endolymphatic hydrops (EH; dotted line).

The displacement transmission ratio (DTR) of the stapes head to the umbo was used to represent the middle ear transfer function under normal and EH conditions in this study.  Figure 6 shows the mean DTR values (*n* = 6) at frequencies from 0.5 to 8 kHz. In the control ears, the displacement of the stapes head was slightly less than that of the umbo by factors of 0.64–0.72 at frequencies above 2 kHz and close to the umbo by factors of 0.9–1.06 at frequencies of 0.5–2 kHz. When EH was present in the cochlea, the displacement of the stapes head was much lower than that in the umbo, by factors of 0.2–0.5, at frequencies of 4–8 kHz. As for frequencies below 2 kHz, a factor of 0.9–1 suggested nearly equal displacement between stapes head and umbo (Figure 6). The lower DTR of the stapes head to the umbo in the EH group than in the control group suggested that vibration of the stapes head was reduced more than that of the umbo by EH.

### 3.5. The Morphology Changes of the EH Model

**Figure 7:**
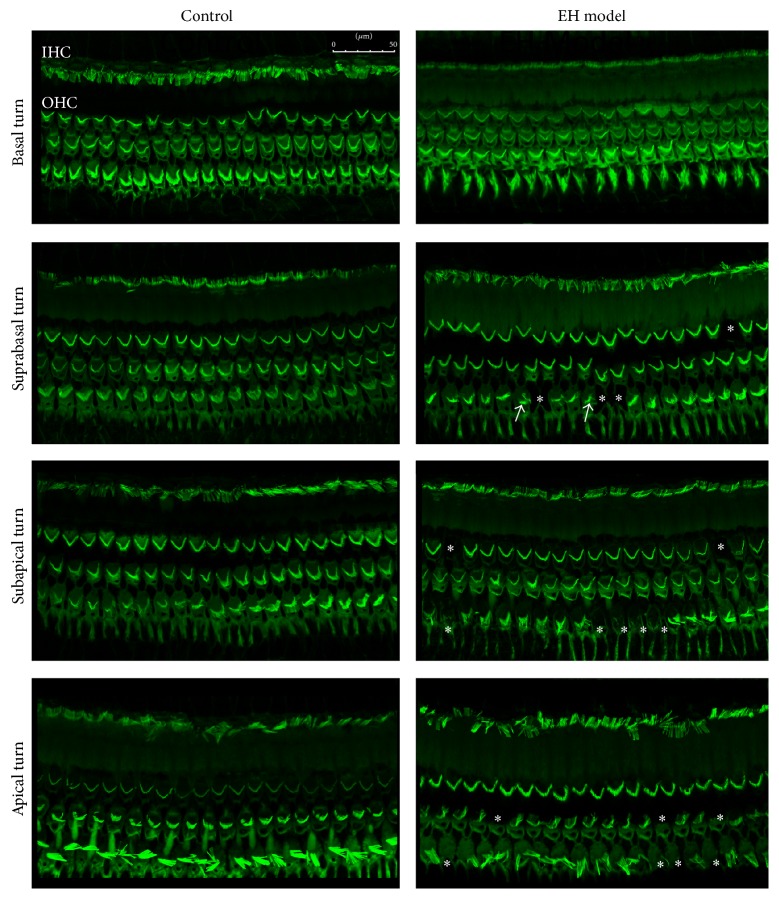
Confocal image of cochlea basilar membrane staining with FITC-conjugated phalloidin in the four turns of control group and EH model group. Accidental loss of stereocilia of outer hair cells (*∗*) in the suprabasal, subapical, and apical turns was observed. Sporadic collapse and derangement of stereocilia (white arrow) were found in the third row of outer hair cells. Inner hair cells showed normal.

Three ears of two groups were dissected and stained with Alexa Fluor 488-conjugated phalloidin for F-actin of stereocilia. The hair cell bundles in each turn were shown to be normal in the control group 8 weeks later in Figure 6. In the EH group, we could observe the accidental loss of the stereocilia of outer hair cells (star in Figure 7) in the suprabasal, subapical, and apical turn. In the basal turn of EH model, the hair cell bundles were normal. Furthermore, the stereocilia in the third row of outer hair cells were sporadically collapsed and deranged in the suprabasal turn. Inner hair cells showed normal in each turn of the EH model. Interestingly, the most obvious morphology change in the apical and subapical turn of the EH group compared to the control group, which showed the high level extent of the labyrinthine hydrops and low frequency ABR threshold shift.

## 4. Discussion

We reported the effect of endolymphatic hydrops of live guinea pigs on the ABR threshold, morphology changes, and movements of the umbo, stapes head, and RWM under 85 dB SPL pure tone stimuli in the external auditory canal in this study. We had explored the mechanism of the hearing loss in EH model, which showed displacements reduction of the umbo, stapes head, and RWM was greater at low frequency (<1 kHz) and the stereocilia of outer hair cell were missing in apical and subapical turn, which was associated with the extent of the endolymphatic hydrops.

### 4.1. Dynamic Properties of the Umbo, Stapes Head, and RWM

Since the target location of laser beam along the TM affects data quality in terms of the signal-to-noise ratio and the TM is directly attached to the ossicular chain (the umbo and the lateral process of the malleus), TM movement in this study was measured on the medial side of the umbo to minimize variability. The umbo displacement was slightly decreased in the EH group compared with the control group at all measured frequencies and the differences were more notable at low frequencies. The biggest reduction in umbo displacement was 5.01 dB (ref = 1 pm) at 0.5 kHz. The control group shared a similar displacement amplitude-frequency curve of umbo with that reported by Guan and Gan [17] (Figure  3a in their paper). But data in this study was generally lower. This discrepancy may be explained by the different measurement sides and the subtle difference between angles of the laser beam in both studies. In the study by Guan and Gan, the target position was at the lateral side of umbo, while in this study the medial side of the umbo was taken as test point. Shinohara [20] and Murakami et al. [21] suggested that a positive inner ear fluid pressure weakened the vibration of the umbo mainly at low frequencies (≤1 kHz). Jang et al. [22] reported that the endolymphatic pressure load caused greater reduction in the umbo velocity than did the perilymphatic pressure load at frequencies below 1 kHz. All these findings supported the result in this study.

The displacement of the incus tip in guinea pigs under 80 dB SPL acoustic stimulation in the ear canal was reported by Guan and Gan [17] and by Chen et al. [23]. The measurement of the stapes head in this study was recorded at a nearby site compared with the incus tip reported previously, both around the incudostapedial joint. The mean stapes head displacement of the control group in this study was in accordance with that reported by Guan and Gan [17] (Figure  4A in their paper) on the whole but was generally higher than that reported by Chen et al. [23]. Murakami et al. [21] reported that the stapes velocity decreased over 0.4–5.0 kHz in fresh human temporal bones with increased hydrostatic pressure of the inner ear under sound pressure of 114 dB SPL. And the effect was less marked at high frequencies than at low frequencies. Lord et al. [24] and Gyo et al. [25] found that the stapes motion changed at low frequencies when inner ear fluid was drained. In this study, the mean displacement amplitude of the stapes in the EH group decreased at all measured frequencies, with profound reduction below 1 kHz. All those findings suggested that cochlear fluid pressure/volume can affect inner ear impedance and cause changes in stapes displacement under acoustic stimulation.

The round window is one of the two openings into the cochlea from the middle ear. The RWM serves as a barrier between the middle ear cavity and the cochlea and plays an important role in the middle ear and cochlear mechanics. The mechanical response of the RWM can be indirect detection of acoustic dysfunction [15]. Compared with literature published by Guan and Gan [17] (Figure  5A in their paper), these two studies shared a parallel mean displacement amplitude-frequency curve and the data were in the same order of magnitude. The data of RWM vibration from ears with EH have not yet been reported. The maximum reduction of the RWM displacement was 13.01 nm (8.5 dB ref = 1 pm) at 500 Hz. The difference between 1 kHz and 4 kHz was about 1–1.8 dB, which is relatively flat. A second difference-peak was found at 6 kHz (5 dB). Those findings suggested that EH has an influence on the vibration of the RWM and that changes occur mainly at low frequencies.

### 4.2. EH Effect on Transfer Function of the Middle Ear

Studies of motion of the ossicular chain revealed that it was more complicated than a simple piston-like motion and that the complexities increased with frequency. In order to evaluate the transfer function of middle ear approximately, DTR of single points (the TM to the footplate/incus tip) was calculated by researchers [17, 26]. Nevertheless, the DTR of the stapes head to the umbo may not reveal the exact middle ear transfer function. Through numerical conversion, the results of the control group from Guan and Gan [17] showed that the mean displacement of the incus tip was less than that of the TM by a factor of 0.2–0.34 at lower frequencies and low to 0.125 at higher frequencies. Results from this study do not correspond well with those reported by Guan and Gan [17]. As described before, the angle between the motion of the umbo and the laser beam in this study is different and this may be one reason for the discrepant findings between the two studies. Moreover, the complex motion of the stapes may increase the interindividual variations in the results of DTR and may underlie differences in the findings. When EH was present in the cochlea, the displacement of the stapes head was much lower than that of the umbo, by factors of 0.2–0.5, at frequencies of 4–8 kHz. The lower DTR in the EH group than in the control group suggested that vibration of the stapes head was reduced more than that of the umbo by EH. Both the umbo and stapes head vibration decreased due to the increased inner ear impedance, decreasing the stapes footplate movement. As the ossicular rotation axis and incudostapedial joint have some laxity, the increased inner ear impedance may not be wholly transmitted to the umbo. Therefore, the reduction of stapes head displacement should be greater, as described by the DTR.

### 4.3. The Endolymphatic Hydrops and Hearing Loss

Since the milestone findings on the temporal bones of Ménière's Disease (MD) patients [4, 27, 28], endolymphatic hydrops (EH) has been considered as the histopathological origin of MD, as characteristic morphological changes were reported previously by surgical obstruction of the endolymphatic sac (ES) in guinea pig [29]. Because surgical experiment induction procedure is still by far the most common method for producing experimental endolymphatic hydrops (EH), it is particularly important to characterize the pathologic changes in this model so that their relevance to Ménière's Disease can be fully appreciated. The simplest explanation for the hearing loss in the endolymphatic hydrops is initial loss of cochlear sensitivity, the shift in the cochlear microphonics (CM), and Distortion Product Otoacoustic Emission (DPOAE); increased SP (summating potential) amplitude, with a reduction in EP (endocochlear potential), is one which has been suggested by researchers who have performed acute endolymph injections [30]. Many authors have demonstrated that the significant correlation between the degree of hydrops and a reduction in the low frequency cochlear microphonic change was found in short- and long-standing surgically induced hydrops guinea pigs [31–33]. Because high-frequency hearing is ultimately affected in both the human and the animal conditions, these same studies imply a poor relationship between hair cell loss and the degree and nature of hearing loss. In our experiment, we have quantified the extent of hydrops in each turn of EH models. The most serious labyrinthine hydrops is in the subapical turn, and the second is in the suprabasal turn. Meanwhile, the mean ABR threshold of the EH cases was significantly higher than that of the control group at 0.5, 1, 2, 4, 6, and 8 kHz frequencies. Strong positive correlation was found between HR at apical turn and ABR threshold elevation at 1 kHz, as well as between HR at subapical turn and ABR threshold elevation at 2 kHz. In order to underline the morphology change of the hair cells stained with Alexa Fluor 488-conjugated phalloidin, we could observe the accidental loss of the outer hair cells and sporadic collapse of the stereocilia in the suprabasal, subapical, and apical turn. In the basal turn of EH model, there were almost normal hair cell bundles. Inner hair cells showed normal morphological features in each turn of the EH model. Interestingly, the most obvious morphology change in the apical and subapical turn of the EH group compared to the control group, which showed the high level extent of the labyrinthine hydrops and low frequency ABR threshold shift. We could not observe the serious hair cell damage in the basal and suprabasal turn with hydrops and higher frequency ABR threshold elevation, which should be needed for long-time EH model observation. Overall, the hearing loss of the surgical EH model in our experiment could be explained for two reasons: one is that the higher inner ear impedance in EH affected the dynamic behavior of the two opening windows of the cochlea and then reduced the vibration of the ossicular chain by increasing the afterload, resulting in acoustic dysfunction. The other is the morphological damage of the stereocilia of the outer hair cells especially in the apical and subapical turn with more serious labyrinthine hydrops.

## 5. Conclusions

In this study, an EH model was created in live guinea pigs by obliteration of the endolymphatic sac. It is the first time to detect the dynamic behaviors of the ossicular chain and the two opening windows by LDV in vivo guinea pigs. EH reduced the displacement of the umbo, stapes, and RWM mainly at lower frequencies. Elevation of the ABR threshold was noted at all measured frequencies. The LDV provides an accurate assessment of the dynamic properties of the middle ear and cochlear mechanics in EH animal models. The displacement of Reissner's membrane caused by EH indicates that the membranous cochlear duct reached a higher hydrostatic pressure. The LDV results suggested that abnormal endolymphatic pressure affects the dynamic behaviors of the two opening windows of the cochlea and that this may be one of the major reasons for hearing loss at lower frequencies in EH-related conditions.

## Figures and Tables

**Table 1:** HR for each turn in the EH models.

Animal number	HR for each turn			
	Apical turn	Subapical turn	Suprabasal turn	Basal turn
1	3.024615	3.167749	2.523004	2.537709
2	2.369168	3.506736	2.762338	1.780297
3	2.284045	1.856286	2.01498	2.967329
4	2.238242	5.37974	5.556607	2.300098
5	2.531883	2.139673	1.747295	2.373561
6	2.753971	4.046874	3.527099	2.790851
Mean ± SD	2.53 ± 0.30	3.34 ± 1.29	3.02 ± 1.38	2.45 ± 0.41

**Table 2:** The ABR threshold of the control group and EH group at the frequency of 0.5, 1, 2, 4, 6, and 8 kHz.

Frequency (Hz)	Before surgery in control	8 weeks after surgery in control	Before surgery in EH	8 weeks after surgery in EH
	Mean ± Std. deviation (dB SPL)			
500	34.17 ± 4.91	35 ± 2.58	34.17 ± 5.84	54.17 ± 3.96
1000	25.8 ± 3.76	26.67 ± 2.1	31.67 ± 4.08	48.33 ± 3.33
2000	25.8 ± 2.04	24.17 ± 0.83	27.5 ± 4.18	49.17 ± 5.07
4000	24.17 ± 3.76	23.33 ± 1.67	25.8 ± 2.04	50 ± 5.32
6000	30.83 ± 5.84	31.67 ± 2.47	28.33 ± 2.58	57.5 ± 6.16
8000	33.33 ± 7.52	30.83 ± 3.75	31.67 ± 2.58	68.33 ± 8.23
